# Engineered MSC-exosomes as CRISPR-Cas9 delivery vectors for precision immunotherapy

**DOI:** 10.3389/fbioe.2026.1823483

**Published:** 2026-08-17

**Authors:** Dandan Guo, Wei Zhang, Wenbo Dou, Yitong Yang, Binbin Sun, Mengyu Qiu, Kai Wang, Jianjun Zhai, Shuying Feng

**Affiliations:** 1 Medical College, Henan University of Chinese Medicine, Zhengzhou, China; 2 Henan Engineering Research Center for Chinese Medicine Foods for Special Medical Purpose, Zhengzhou, China

**Keywords:** autoimmune diseases, clinical translation, CRISPR-Cas9, gene editing, immunotherapy, mesenchymal stem cell-derived exosomes, targeted delivery, vehicle engineering

## Abstract

Mesenchymal stem cell-derived exosomes (MSC-Exos) combined with CRISPR-Cas9 hold substantial therapeutic potential for immune-mediated diseases, although significant technical obstacles remain. This review presents a comprehensive analysis of strategies for engineering MSC-Exos as delivery vehicles for CRISPR-Cas9, with emphasis on cargo-loading methodologies and surface modifications that enable targeting specific immune cell populations. We examine the mechanistic basis for the therapeutic effects of these engineered platforms and critically assess the challenges impeding clinical translation, including manufacturing scalability, safety concerns, and regulatory considerations. Key areas of focus for this Research Topic—vehicle engineering, cargo packaging efficiency, biological barriers, and *in vivo* safety—are systematically addressed. Finally, we discuss emerging directions, including next-generation gene editors and stimulus-responsive biomaterials. This review provides a balanced framework for advancing MSC-Exos-based nanoplatforms toward precision gene therapies for immune disorders.

## 1 Introduction

Immune-mediated diseases, including autoimmune disorders, chronic inflammatory conditions, and transplant rejection, pose significant clinical challenges due to their complex etiology and the limited efficacy of conventional immunosuppressive therapies (Kalluri and LeBleu, 2020; Herrmann et al., 2021; Jinek et al., 2012; Li et al., 2018; Rosenblum et al., 2012). The emergence of cell-based and gene-based therapeutics has opened promising new avenues. However, despite the promise of MSC-Exos, the clinical translation of MSC transplantation faces practical hurdles, such as poor engraftment efficiency, limited *in vivo* persistence, and potential safety risks (Galipeau and Sensébé, 2018).

A paradigm shift has occurred with the realization that the therapeutic benefits of MSCs are largely mediated by their secreted extracellular vesicles, particularly exosomes (MSC-Exos) (Wu et al., 2019). These nanoscale vesicles carry immunomodulatory cargo from their parent cells and offer greater stability, lower immunogenicity, and improved storage (Wang et al., 2021). However, it is paramount to note that the native tropism of MSC-Exos does not inherently exhibit specificity for pathogenic immune cells, thereby necessitating extensive engineering interventions to achieve precise targeting. Concurrently, the CRISPR-Cas9 system has heralded a transformative era in precision medicine by enabling direct correction of genetic defects underlying various diseases (Doudna and Charpentier, 2014). Nonetheless, its clinical application is severely hampered by the lack of safe and efficient *in vivo* delivery vectors capable of targeting specific immune cell populations (Doudna and Charpentier, 2014).

Conventional delivery systems, such as viral vectors and synthetic nanoparticles, face significant challenges, including immunogenicity, limited packaging capacity, and poor target-cell specificity (Galipeau and Sensébé, 2018). Although synthetic nanoparticles offer advantages in scalability, they are often limited by rapid clearance, poor cell-type selectivity, and potential cytotoxicity. In contrast, MSC-Exos, as naturally evolved nanocarriers, provide innate biocompatibility, barrier-crossing ability, and engineerable surfaces for targeted delivery (Sterzenbach et al., 2017). Nevertheless, translating engineered exosomes into clinical and practical applications encounters a unique array of challenges, encompassing scalable manufacturing, batch-to-batch variability, and potential immunogenicity arising from surface modifications. Specifically, batch-to-batch variability of engineered MSC-Exos most commonly arises from fluctuations in particle size distribution, cargo loading efficiency (e.g., Cas9/sgRNA content), surface ligand density, and targeting or editing efficiency, all of which are highly sensitive to MSC source, culture conditions, and isolation methods. This review elucidates the utilization of engineered MSC-Exos as intelligent vectors for delivering CRISPR-Cas9 to modulate the immune system. We offer an in-depth analysis of current engineering approaches, investigate the synergistic mechanistic actions of this combined system, and critically evaluate its translational trajectory, prospective advancements, and the substantial obstacles that persist. Throughout the manuscript, we distinguish engineering strategies that are currently technically feasible from those that remain aspirational, evaluating approaches based on translational readiness—including cargo loading efficiency, functional editing outcomes, manufacturing reproducibility, and *in vivo* validation status.

Unlike previous reviews that primarily focus on either exosome biology or CRISPR delivery technologies in isolation, this review provides an integrated, decision-oriented framework specifically for MSC-Exos-based CRISPR-Cas9 systems. We systematically compare various engineering strategies—including cargo loading, surface targeting, and hybrid systems—using translational readiness as the primary evaluation criterion, considering not only editing efficacy but also manufacturing scalability, safety profiles, and regulatory feasibility. By explicitly distinguishing approaches that are currently technically feasible from those that remain speculative, we aim to offer actionable guidance for researchers and clinicians navigating this rapidly evolving field. Consistent with the focus of this Research Topic, we herein synthesize current knowledge on engineering MSC-Exos for CRISPR-Cas9 delivery. Our discussion encompasses five principal areas emphasized in the collection: (i) vehicle engineering for enhanced targeting specificity; (ii) cargo packaging approaches; (iii) mechanisms for overcoming biological delivery barriers; (iv) safety considerations, including off-target effects; and (v) challenges associated with clinical translation and scalable manufacturing. This structured analysis is intended to inform future development of exosome-based platforms for precision immune modulation.

## 2 Biogenesis and immunomodulatory functions of MSC-Exos

MSCs are multipotent stromal cells with self-renewal capacity and the potential to differentiate into diverse cell lineages. They are naturally found in various tissues, including bone marrow, adipose tissue, and umbilical cord (Phinney and Pittenger, 2017). Initially, their therapeutic effects were attributed to direct differentiation and replacement of damaged cells. It is now well-established that paracrine signaling, mainly via secreted extracellular vesicles (EVs), particularly exosomes, is the primary mechanism of MSC therapy (Uccelli et al., 2008). In this context, several authoritative guidelines, such as the MISEV2018 recommendations, emphasize standardized characterization of extracellular vesicles, including size distribution, protein markers, purity, and functional potency, which are particularly critical when exosomes are engineered as gene-editing delivery platforms.

MSC-Exos represent a category of nanoscale extracellular vesicles (30–150 nm in diameter) characterized by a phospholipid bilayer. Their biogenesis occurs through the endosomal pathway, and their lumen is enriched in biomolecules, including proteins, nucleic acids, and lipids (Rani et al., 2015). They function as key mediators of intercellular communication by transferring bioactive molecules to recipient cells (van den Boorn et al., 2013). Critically, as a cell-free alternative, MSC-Exos circumvent the risks associated with whole-cell transplantation, such as vascular occlusion and tumorigenicity, while simultaneously enhancing batch-to-batch consistency and storage flexibility (Berebichez-Fridman and Montero-Olvera, 2018). However, it should be noted that achieving true batch-to-batch consistency in engineered exosomes remains a significant manufacturing challenge.

The immunomodulatory potency of MSC-Exos is demonstrated by their regulatory effects on both innate and adaptive immunity (Théry et al., 2018). Within the innate immune system, they potently shift macrophage polarization from a pro-inflammatory M1 phenotype toward an anti-inflammatory, tissue-reparative M2 state (Harrell et al., 2019). This reprogramming is achieved by downregulating M1 markers and cytokines (e.g., CD86, iNOS, TNF-α, IL-6) while upregulating M2 markers and mediators (e.g., CD206, Arg-1, IL-10, TGF-β) (Valadi et al., 2007). Signaling pathways such as JAK-STAT and NF-κB are critically involved in this phenotypic switch (Kordelas et al., 2014). Moreover, specific miRNAs packaged within MSC-Exos, like miR-146a, can suppress Toll-like receptor (TLR) signaling and NLRP3 inflammasome activation, thereby dampening inflammation (Wang T. et al., 2020). However, the precise composition of miRNA cargo can vary with MSC source and culture conditions, potentially affecting functional consistency.

In adaptive immunity, MSC-Exos modulate dendritic cell (DC) maturation, reducing their surface expression of co-stimulatory molecules (CD80, CD86, CD40) and impairing their ability to activate naïve T cells (Lo Sicco et al., 2017). They directly suppress T cell proliferation and pro-inflammatory cytokine production while promoting the expansion of regulatory T cells (Tregs) (Reis et al., 2018). Regarding B cells, *in vitro* studies have shown that MSC-Exos inhibit plasma cell differentiation and antibody production, while fostering the development of regulatory B cells (Bregs) (Di Trapani et al., 2016). These multifaceted actions, as demonstrated in both *in vitro* and *in vivo* studies, establish MSC-Exos as powerful endogenous regulators of immune homeostasis, providing a compelling rationale for their use as vectors for precision immunomodulation (Elashiry et al., 2020). Nevertheless, their inherent pleiotropic effects necessitate careful engineering to ensure therapeutic specificity and avoid unintended immunosuppression. For example, excessive or non-specific immunosuppression may increase susceptibility to opportunistic infections or impair immune surveillance against malignant cells, highlighting the need for precise targeting and controlled gene-editing activity.

## 3 CRISPR-Cas9 system: Mechanism and delivery challenges

The CRISPR-Cas9 system, derived from a prokaryotic adaptive immune mechanism, has been repurposed as a highly versatile genome-editing tool (Wiklander et al., 2015). The system relies on two key elements: the Cas9 endonuclease, which introduces DNA double-strand breaks (DSBs), and a single-guide RNA (sgRNA) that directs Cas9 to a specific genomic sequence by recognizing a protospacer adjacent motif (PAM) (Gee et al., 2020). Following the cleavage, the cell primarily employs two distinct repair mechanisms: non-homologous end joining (NHEJ), an error-prone process frequently used for gene disruption, and homology-directed repair (HDR), which facilitates precise gene correction or insertion in the presence of a donor DNA template (Hryhorowicz et al., 2017).

Despite its transformative potential, the therapeutic application of CRISPR-Cas9 *in vivo* is critically dependent on the delivery vehicle (Jiang and Doudna, 2017). An ideal vector must encapsulate and protect the often large and negatively charged CRISPR-Cas9 components (plasmid DNA, mRNA, or ribonucleoprotein (RNP)), facilitate efficient cellular uptake, enable intracellular release, and minimize off-target effects and immune reactions (Wan et al., 2022).

Viral vectors, particularly adeno-associated viruses (AAVs), are efficient but suffer from limited packaging capacity (<5 kb), pre-existing immunity in patients, and potential risks of insertional mutagenesis (Newby and Liu, 2021). Non-viral vectors, such as lipid nanoparticles (LNPs) and polymeric nanoparticles, offer improved safety profiles and scalability. However, their delivery efficiency can vary substantially depending on the target cell type, and they often face challenges related to cell-type selectivity and rapid clearance by the mononuclear phagocyte system (Wang D. et al., 2020). Notably, rapid clearance by the mononuclear phagocyte system represents a shared challenge for both LNPs and exosome-based delivery systems (Wiklander et al., 2015). Furthermore, the surface engineering options for LNPs are relatively limited compared to exosomes, as LNPs lack the abundant surface proteins that enable diverse genetic and chemical conjugation strategies. In addition, the immunogenicity and potential toxicity of certain LNP formulations remain important safety concerns that require careful consideration in clinical translation (Mitchell et al., 2021). Therefore, developing a delivery platform that combines the high efficiency of viral vectors with the safety and flexibility of synthetic systems is a paramount objective (Mitchell et al., 2021). MSC-Exos, with their natural origin and high engineerability—owing to the presence of surface proteins amenable to both genetic and chemical modification—emerge as a highly promising candidate to bridge this critical gap (Elsharkasy et al., 2025), though their loading capacity for large genetic payloads still requires thorough evaluation. To systematically illustrate the comparative advantages of MSC-Exos over conventional delivery systems, we summarize the key performance metrics of various platforms in Table 1.

**TABLE 1:** Comparison of MSC-Exos with conventional nanocarriers for CRISPR delivery.

Delivery system	Biocompatibility	Targeting capability	Cargo capacity	Immunogenicity	Scalability	Ref.
Viral vectors (AAV)	Moderate	Low (serotype-dependent)	Limited (<5 kb)	High	Difficult	Newby and Liu (2021)
Lipid nanoparticles (LNPs)	Moderate	Moderate (via modification)	High	Moderate	High	Wang D. et al. (2020)
Polymeric nanoparticles	Moderate	Moderate (via conjugation)	High	Variable	High	Mitchell et al. (2021)
MSC-Exos (naïve)	High	Low (innate tropism only)	Moderate	Low	Moderate	Sterzenbach et al. (2017)
Engineered MSC-Exos	High	High (via surface engineering)	Moderate-High	Low-Moderate	Moderate	Present review

From a nanotherapeutic perspective, MSC-Exos represent a unique class of biologically derived nanoparticles that bridge the gap between synthetic nanocarriers and cell-based therapies. Compared to fully synthetic systems, they offer inherent biofunctionality and immune compatibility; compared to viral vectors, they provide greater safety and greater flexibility for surface engineering. This positions engineered MSC-Exos as a versatile nanoplatform for targeted genome editing, with the potential to combine the best of both synthetic and biological worlds.

## 4 Engineering MSC-exos-CRISPR/Cas9 delivery: strategies and applications

**FIGURE 1:**
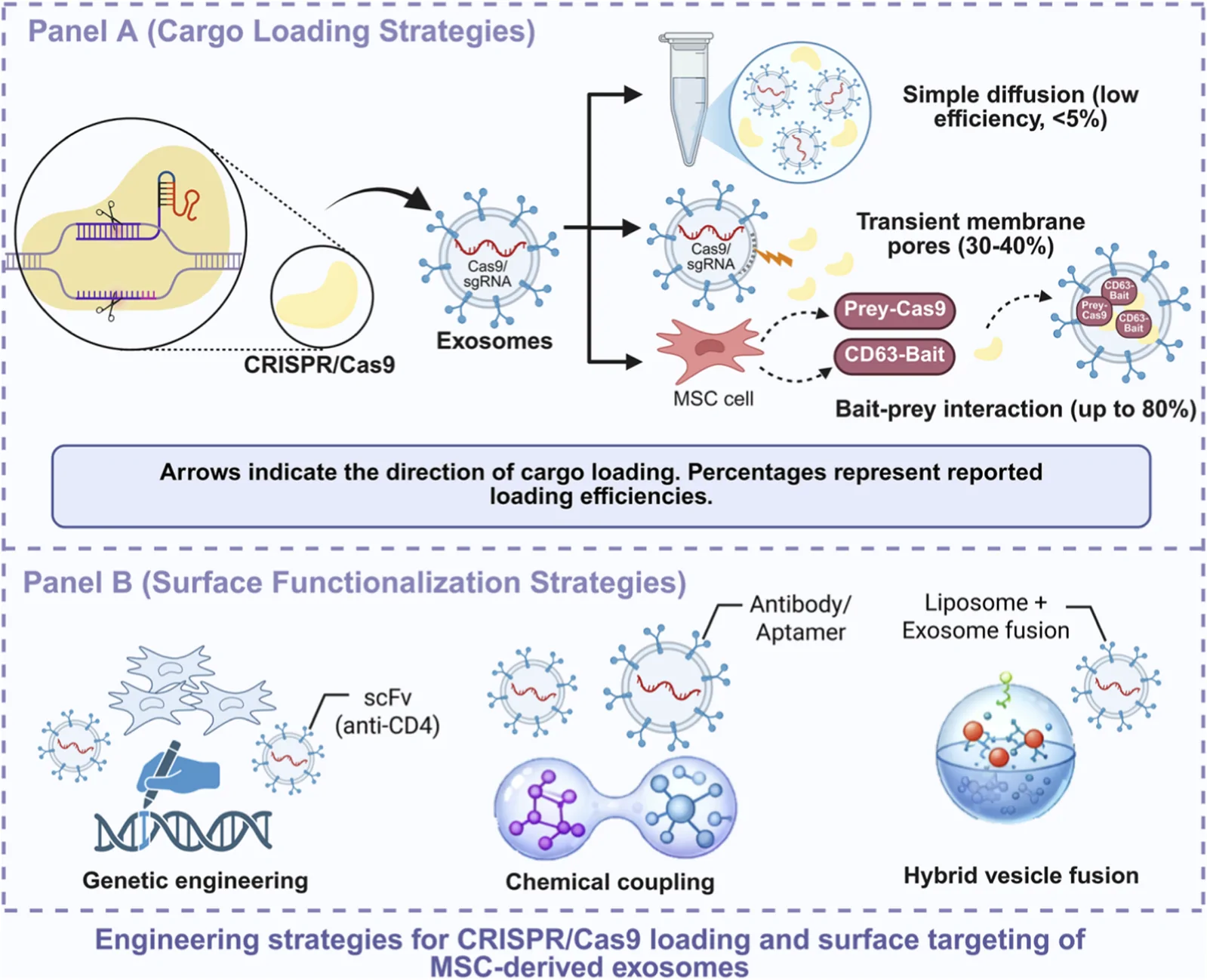
Engineering strategies for CRISPR/Cas9 loading and surface targeting of MSC-derived exosomes. Panel **(A)** Cargo loading strategies: (i) Passive loading relies on simple diffusion (ii) Physical loading (e.g., electroporation). creates transient membrane pores. (iii) Active loading (Affinity Capture) genetically engineers parent MSCs to co-express a “bait” protein on exosomal membranes and a Cas9-“prey” fusion for high-efficiency, specific packaging during biogenesis. Panel **(B)** Surface functionalization strategies: (i) Genetic engineering enables endogenous display of targeting ligands. (ii) Chemical conjugation allows post-isolation attachment of targeting moieties. (iii) Hybrid vesicle fusion combines MSC-Exos with functionalized synthetic liposomes. Loading efficiencies for different methods are indicated as follows: passive loading (<5%), physical loading (30%–40%), and active loading via bait-prey interaction (up to 80%).

The development of engineered MSC-Exos as delivery vehicles for CRISPR-Cas9 involves two complementary engineering objectives: efficient cargo loading and targeted delivery. The workflow for transforming naïve MSC-Exos into precision vectors is conceptually illustrated in Figure 1, detailing the key steps from cargo loading to surface functionalization.

### 4.1 Cargo loading strategies

The effective delivery of CRISPR-Cas9 relies on the efficient encapsulation of its functional components within MSC-Exos. As depicted in Figure 1A, prevailing cargo-loading methodologies can be categorized into three tiers, reflecting a progression in technical sophistication (Lener et al., 2015). While passive incubation is straightforward, it yields low encapsulation efficiency and poor control over cargo dosage, rendering it unsuitable for most therapeutic applications (Kooijmans et al., 2016). Physical methods, such as electroporation, can improve loading efficiency *in vitro* but often compromise vesicle integrity and induce cargo aggregation, potentially affecting functionality and safety (Hood, 2016). Using a reversible heterodimerization-based loading strategy, Osteikoetxea et al. (2022) reported that engineered EVs could be loaded with approximately 25 Cas9 molecules per vesicle, achieving up to 51% gene editing in reporter cells and 6% indel efficiency at the PCSK9 locus, providing direct evidence for CRISPR-Cas9 RNP loading and functional delivery (Osteikoetxea et al., 2022). The most promising approach for clinical translation is active loading, particularly the “affinity capture” method. This strategy genetically engineers parent MSCs to co-express a “bait” protein (e.g., fused to the exosomal transmembrane protein CD63) and the Cas9 protein fused to a cognate “prey” domain. During exosome biogenesis, the Cas9-'prey’ complex is actively recruited and packaged into the forming vesicles via high-affinity interactions, achieving high loading efficiency and specificity while preserving exosomal function. This strategy, conceptually demonstrated by the EXPLORs system for protein loading (Yim et al., 2016), has since been adapted for CRISPR-Cas9 RNP packaging (Osteikoetxea et al., 2022). A critical consideration for this approach, however, is the genetic manipulation of the parent cells, which introduces regulatory complexities. Furthermore, the loading efficiency for larger editors, such as prime editors, remains to be fully optimized.

Although affinity capture enables efficient loading of Cas9, the larger molecular weight of next-generation editors—such as prime editors—poses packaging challenges for MSC-Exos. Emerging strategies to address this limitation include split-intein systems for post-delivery editor assembly and optimized bait-prey designs with enhanced affinity. These advances are essential for extending exosome-based delivery beyond Cas9 to encompass a broader spectrum of genome-editing tools.

### 4.2 Precision surface functionalization for targeted delivery

Achieving precision in “Targeted Therapeutics” begins with efficient cargo loading, while the ultimate key lies in delivering genome editors to specific immune subsets with high specificity. Targeted delivery to specific immune cell subsets is therefore paramount. To this end, surface engineering equips MSC-Exos with molecular guidance systems, as depicted in Figure 1B (Armstrong et al., 2017). Genetic engineering of parent cells facilitates the endogenous display of targeting ligands (e.g., single-chain variable fragments, scFvs, peptides) fused to exosomal membrane proteins (e.g., lysosome-associated membrane protein 2B), thereby ensuring stable, correctly oriented presentation (Alvarez-Erviti et al., 2011). As a more modular alternative, chemical conjugation via bioorthogonal chemistry (e.g., click chemistry) allows for precise post-isolation attachment of targeting moieties (e.g., antibodies, aptamers) without genetic manipulation (Smyth et al., 2014). Furthermore, the creation of hybrid vesicles by fusing MSC-Exos with functionalized synthetic liposomes represents a powerful synergy, combining the innate biological properties of MSC-Exos with the tunable surface chemistry of synthetic systems (Liu et al., 2022; Dou et al., 2026).

It is crucial to recognise that these modifications can potentially alter the pharmacokinetics and immunogenic profile of exosomes. The density and orientation of targeting ligands are critical determinants of efficacy but remain challenging to control with high precision. Additionally, the long-term stability of chemically conjugated groups *in vivo* necessitates thorough investigation. Rather than treating these engineering strategies as equivalent alternatives, their relative merits should be evaluated using decision-relevant metrics, including loading yield, cargo integrity, functional editing efficiency, batch-to-batch reproducibility, and regulatory burden. To systematically compare the advantages and limitations of the primary engineering strategies discussed above, we provide a consolidated summary in Table 2.

**TABLE 2:** Strategic engineering approaches for MSC-Exosome-CRISPR-Cas9 delivery.

Strategy	Core principle	Advantages	Limitations	Ref.
Affinity loading	“Bait-prey” interaction-mediated co-compartmentalization during exosome biogenesis	High loading efficiency; minimal vesicle disruption; high cargo specificity	Requires viral transduction; ATMP regulatory burden; limited capacity for large editors	Yim et al. (2016)
Surface display	Genetically encoded ligand fusion to exosomal membrane proteins	Durable *in vivo* targeting; scalable production; stable ligand orientation	Uncontrolled ligand density; altered surface proteome	Alvarez-Erviti et al. (2011)
Hybrid fusion	Fusion with synthetic liposomes pre-loaded with CRISPR-Cas9	Increased cargo capacity; combines biological and synthetic features; no genetic modification	Batch heterogeneity; accelerated plasma clearance	Dou et al. (2026)
Chemical conjugation	Covalent coupling of targeting moieties post-exosome isolation	Rapid, modular; simplified regulatory pathway; broad ligand compatibility	Suboptimal coupling yield; ligand instability *in vivo*	Smyth et al. (2014)

These surface engineering strategies collectively transform MSC-Exos from passive delivery vehicles into intelligent, cell-type-specific nanoplatforms for targeted genome editing.

For instance, anti-CD4 single-chain variable fragments (scFvs) displayed on exosome surfaces have been employed to direct CRISPR-Cas9 to CD4^+^ T cells, offering a strategy for HIV reservoir elimination (Campbell et al., 2019). In autoimmune disease models, exosomes decorated with anti-CD19 antibodies facilitated selective delivery to B cells, enabling targeted modulation of pathogenic antibody production (Cheng et al., 2018). For macrophage-targeted immunotherapy, mannose-modified MSC-Exos have been utilized to deliver anti-inflammatory cargo to M1-type macrophages in colitis models, promoting polarization toward the M2 phenotype (Duan et al., 2021). Additionally, targeting ligands such as RGD peptides have been used to home engineered exosomes to inflamed tissues via integrin recognition, as demonstrated in arthritis models (Kim et al., 2021). These examples illustrate the versatility of surface engineering for achieving disease-relevant immune cell specificity.

### 4.3 Biological barriers to intracellular delivery

Effective CRISPR-Cas9 editing requires that engineered MSC-Exos navigate multiple biological barriers following administration. These include rapid clearance by phagocytic cells, limited accumulation at inflamed tissue sites, and—most significantly—inefficient release from endosomal compartments after cellular internalization (Mitchell et al., 2021). A substantial proportion of internalized exosomes undergo lysosomal degradation, restricting cytosolic access of Cas9 cargo and limiting editing efficiency.

Recent engineering efforts have focused on incorporating endosome-disrupting functionalities into exosome surfaces or hybrid vesicle formulations. Fusogenic peptides derived from viral proteins, including influenza hemagglutinin, promote membrane fusion and cargo release in response to endosomal pH (Yim et al., 2016). Synthetic pH-responsive polymers such as GALA and KALA undergo conformational transitions in acidic environments, disrupting endosomal membranes and facilitating cytosolic delivery (Smyth et al., 2014). Despite their potential, the clinical translation of fusogenic peptides and synthetic pH-responsive polymers faces regulatory hurdles, as these components are derived from viral proteins or synthetic materials that may raise safety concerns regarding immunogenicity and off-target membrane disruption, requiring extensive preclinical evaluation for regulatory approval (Herrmann et al., 2021; Mitchell et al., 2021). These agents can be conjugated to exosome surfaces via click chemistry or expressed as fusion proteins with exosomal membrane scaffolds, enhancing CRISPR-Cas9 editing efficiency in immune cells that are typically resistant to transfection.

Future iterations of this technology may combine endosomal escape capabilities with targeting ligands, yielding multifunctional carriers capable of traversing the entire delivery pathway—from systemic circulation to cytosolic payload release. Such integrated approaches to overcoming biological barriers align with the objectives of this Research Topic.

## 5 Mechanism MSC-exos-CRISPR/Cas9 in immune diseases

### 5.1 Innate immune reprogramming

**FIGURE 2:**
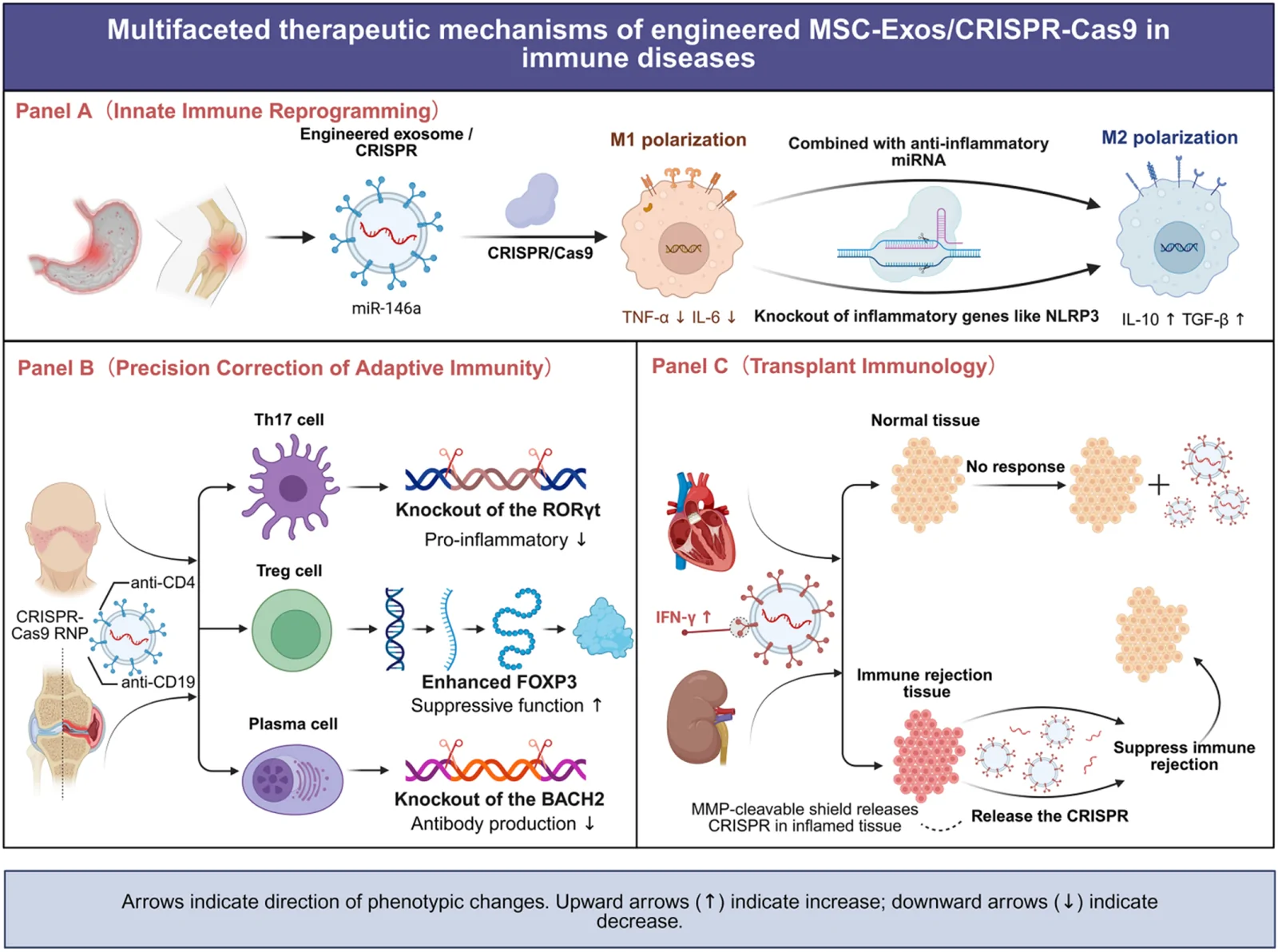
Multifaceted therapeutic mechanisms of engineered MSC-Exos/CRISPR-Cas9 in immune diseases. **(A)** Innate immune reprogramming. Targeting of tissue-resident macrophages with CRISPR-Cas9 ribonucleoprotein (RNP) directed against key inflammatory regulators NLRP3 and MYD88, promoting polarization from pro-inflammatory M1 to anti-inflammatory M2 phenotype, accompanied by cytokine profile shift (TNF-α, IL-6↓; IL-10, TGF-β↑). **(B)** Precision correction of adaptive immunity. Cell-specific editing of pathogenic lymphocyte subsets: Th17 cells: Knockout of RORγt to attenuate pro-inflammatory responses; Tregs: Enhancement of FOXP3 to boost regulatory function; B cells: Targeting of BACH2 to modulate antibody production. **(C)** Transplant immunology and smart switching. Implementation of an IFN-γ sensor enabling conditional immunosuppression upon detection of inflammatory signals associated with graft rejection, minimizing systemic side effects. The engineered MSC-Exos/CRISPR-Cas9 platform exerts its therapeutic effects through a sophisticated, multi-layered mechanism.

As illustrated in Figure 2A, this platform can fundamentally reshape the inflammatory microenvironment by synergising the innate immunomodulatory properties of MSC-Exos with precise CRISPR-Cas9 intervention. For instance, in preclinical mouse models of colitis or arthritis, MSC-Exos engineered to target macrophages deliver CRISPR-Cas9 RNP to key nodes such as Nlrp3 or Myd88 (Campbell et al., 2019), providing *in vivo* evidence. The concurrent administration of exosomes’ intrinsically anti-inflammatory miRNAs, such as miR-146a, along with CRISPR-mediated gene knockout, exerts a potent synergistic impact, leading to long-lasting suppression of IL-1β and IL-18 production and disrupting the vicious cycle of chronic inflammation (Duan et al., 2021). The potential for unintended modulation of other innate immune cells, such as neutrophils, by these engineered exosomes has been raised in *vitro* studies and warrants further investigation *in vivo*. It should be noted that the majority of these findings are derived from *in vitro* systems and small-animal disease models, whereas systematic *in vivo* benchmarking across diverse inflammatory disease models and clinically relevant dosing regimens is still lacking. However, editing efficiency, cell-type selectivity, and the durability of immunomodulatory effects remain highly context-dependent.

### 5.2 Precision correction of adaptive immunity

The platform’s precision is fully unleashed in autoimmune diseases, enabling cell-specific editing within the adaptive immune system (Figure 2B) (Veerman et al., 2019). By decorating MSC-Exos with ligands for lineage-specific markers CD4 and CD19, CRISPR-Cas9 is directed to pathogenic lymphocyte subsets (Kim et al., 2021). This facilitates the selective knockout of master transcription factors that drive immunological responses, such as RAR-related orphan receptor in Th17 cells or BTB and CNC homology 2 in B cells, while sparing bystander immune cells, thereby minimising systemic immunosuppression (Cheng et al., 2018). Furthermore, the platform can be designed to actively induce immune tolerance, for example, by editing the forkhead box P3 (FOXP3) locus to enhance the stability and function of Tregs (Jang et al., 2019). FOXP3 is a lineage-defining transcription factor that governs the development, phenotypic stability, and suppressive function of regulatory T cells, and sustained FOXP3 expression is essential for the maintenance of long-term immune tolerance.

The long-term stability of these edited cell populations and the risk of compensatory pathways emerging are critical unknowns. While these strategies illustrate the potential for highly specific genome editing within adaptive immune cell subsets, current evidence is largely limited to *in vitro* studies and early-stage *in vivo* models. Critical questions regarding long-term lineage stability, compensatory immune pathways, and the robustness of therapeutic effects across heterogeneous disease contexts remain unresolved, underscoring the need for longitudinal *in vivo* validation.

### 5.3 Dual role in transplant immunology

The immunomodulatory role of MSC-Exos in transplant rejection is context-dependent, but engineering strategies can program their function for desired outcomes (Figure 2C) (Honaker et al., 2020). While they can exert potent immunosuppressive effects, such as in preclinical models of Graft-versus-host disease (GvHD) (Valadi et al., 2007), they may potentially enhance immune activation under certain conditions, as suggested by *in vitro* findings (Elashiry et al., 2020). Engineering provides a strategy to direct MSC-Exos towards a stable immunosuppressive phenotype. For instance, this can be achieved by loading them with CRISPR-Cas9 tools designed to delete co-stimulatory molecules in antigen-presenting cells, or by integrating ‘smart’ switches that specifically release immunosuppressive cargo in response to heightened local concentrations of IFN-γ at the rejection site.

## 6 Discussion

Translation of engineered MSC-Exos for CRISPR-Cas9 delivery requires addressing challenges beyond demonstrating efficacy. As highlighted in this Research Topic, scalable manufacturing, comprehensive safety assessment, and regulatory pathway navigation represent critical areas where standardized approaches remain under development. The clinical translation of engineered MSC-Exos faces several systemic hurdles that extend beyond technical proof-of-concept. Developing scalable GMP-compliant manufacturing processes represents a primary obstacle (Anzalone et al., 2019)—an issue central to this Research Topic. This necessitates moving beyond traditional methods, such as ultracentrifugation, to scalable isolation technologies, including tangential flow filtration and chromatography. However, even these advanced methods must contend with the inherent heterogeneity of exosome populations, making batch consistency and definitive characterization (size, titer, purity, potency, and cargo loading efficiency) non-trivial tasks (Wang et al., 2025). Regulatory agencies have yet to establish clear guidelines for such complex cell-free gene therapy products, necessitating early engagement to delineate a clinical approval pathway, which will likely involve rigorous preclinical safety, biodistribution, and off-target assessments (Busatto et al., 2018). From a translational perspective, safety evaluation of MSC-Exos/CRISPR platforms should be structured around four core risk domains: (i) biodistribution and unintended systemic uptake, with particular attention to accumulation in non-target organs such as the liver, spleen, and lungs (Wiklander et al., 2015); (ii) off-target genome editing and Cas-associated immunogenicity, which may provoke chronic immune responses or unintended genomic rearrangements (Charlesworth et al., 2019); (iii) unintended immunomodulation arising from exosomal pleiotropy, including the risk of excessive immunosuppression that could increase susceptibility to infections or impair anti-tumor surveillance (Kalluri and LeBleu, 2020); and (iv) heterogeneity of MSC sources and long-term safety, as exosomal composition and functional potency vary substantially depending on tissue origin (bone marrow, adipose, or umbilical cord), complicating batch consistency and requiring extensive longitudinal validation (Phinney and Pittenger, 2017). These dimensions should serve as primary decision criteria during platform selection and preclinical design.

Notably, the choice of engineering strategy directly impacts translational feasibility and regulatory positioning. Active loading, while efficient, requires genetic modification of parent MSCs, which, from a regulatory perspective, places the product closer to an advanced therapy medicinal product (ATMP), introducing significant complexity. In contrast, hybrid vesicles or post-isolation chemical modification might offer a more modular path but require exhaustive validation to ensure the consistency and stability of the final product. These trade-offs between efficacy, manufacturing complexity, and regulatory burden must be strategically evaluated in the early stages of platform development. Importantly, it should be emphasized that the majority of current studies in this area remain at the proof-of-concept or preclinical stage, with limited validation in large animal models or clinical settings.

In addition to engineering strategies, the route of administration plays a critical role in the therapeutic efficacy and safety of MSC-Exos-based CRISPR delivery. Systemic administration, such as intravenous injection, offers the advantage of broad biodistribution but is associated with rapid clearance by the reticuloendothelial system and limited accumulation at target sites (Wiklander et al., 2015). Local administration routes, including intrathecal, intraperitoneal, and subcutaneous injection, may enhance local bioavailability and reduce systemic exposure, but their applicability depends on the disease target and tissue accessibility (Herrmann et al., 2021). Thus, the choice of administration route should be carefully evaluated in preclinical studies, as it directly influences exosome biodistribution, targeting efficiency, and ultimately clinical translation.

The design of MMP-cleavable shields builds on a growing body of evidence demonstrating the feasibility of enzyme-responsive extracellular vesicle (EV) engineering. For instance, Li et al. (2023) conjugated functional moieties to bacterial outer membrane vesicles (OMVs) via an MMP-cleavable linker, creating a “dynamic shield” that could be specifically removed in the tumor microenvironment to trigger cargo release and enhance therapeutic efficacy. Similar MMP-responsive EV platforms have been designed for targeted cancer immunotherapy, further validating this engineering strategy (You et al., 2025). Notably, MSC-derived exosomes are known to naturally carry and transfer functional MMPs (e.g., MMP-2) to recipient cells, providing an additional layer of rationale for engineering MMP-responsive release in MSC-Exos-based systems. Furthermore, recent evidence demonstrates that naked RNAs can be spontaneously internalized by cells without requiring vesicular encapsulation, provided that extracellular ribonuclease activity is inhibited (Castellano et al., 2025). However, while the MMP-cleavable shield concept is supported by these studies, the precise efficiency of cellular uptake for the released CRISPR-Cas9 RNP from such a system, compared to conventional EV-mediated delivery, warrants systematic experimental validation in future work. The strategy does not rely on efficient uptake of free RNP into the cytosol, but rather positions the EV to release its payload in the disease microenvironment, where the released cargos may be taken up by neighboring cells through various endocytic pathways, albeit with potentially lower efficiency than vesicle-mediated delivery. However, these increasingly sophisticated designs also highlight a fundamental trade-off between editing efficiency and safety: while tighter spatial and contextual control can reduce off-target effects and unintended immune perturbations, it may simultaneously compromise delivery efficiency or editing potency, underscoring the need for balanced optimization in clinical translation. Importantly, the translational justification of such logic-gated designs will require rigorous *in vivo* evidence, including quantitative biodistribution analyses, confirmation of target engagement, and longitudinal safety assessments across multiple disease models, rather than relying on conceptual precision alone. Such designs minimize off-target delivery and enhance therapeutic specificity, addressing a key limitation of current non-viral systems. The successful *in vivo* implementation of these complex logic gates, however, presents a significant bioengineering challenge.

**FIGURE 3:**
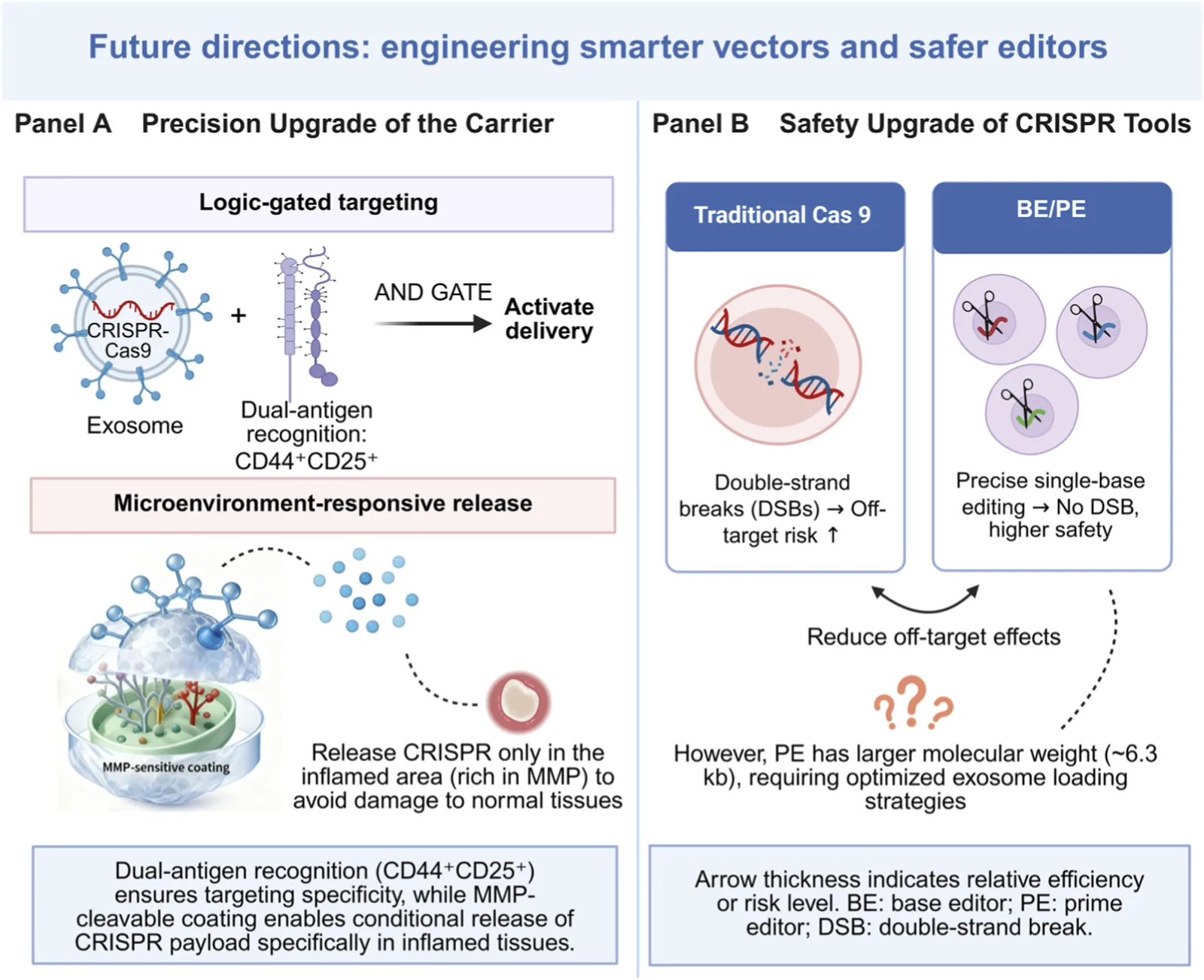
Future directions: engineering smarter vectors and safer editors. **(A)** Precision upgrade of the carrier: strategies for enhancing targeting specificity and safety, including logic-gated targeting based on dual-antigen recognition (e.g., CD44 and CD25) and microenvironment-responsive release utilizing disease-specific protease (e.g., MMP)-cleavable coatings. **(B)** Safety upgrade of CRISPR tools: transition from traditional Cas9, which induces double-strand breaks (DSBs) and carries a higher risk of off-target effects, to more precise base editors (BEs) and prime editors (PEs) that enable single-base changes without DSBs.

Future directions for engineering smarter vectors and safer editors are summarized in Figure 3. Computational and artificial intelligence approaches are increasingly being applied to the design of exosome-based delivery systems. Machine learning models trained on biodistribution data may enable the prediction of optimal surface ligand combinations for targeting specific immune populations. Similarly, AI-driven sgRNA design tools can minimize off-target effects by predicting genome-wide specificity. Deep learning algorithms have also shown promise in predicting exosome tropism from surface protein signatures, potentially accelerating selection of appropriate targeting moieties. These computational strategies, identified in this Research Topic as an emerging frontier, offer opportunities to enhance both efficacy and safety of engineered exosome platforms.

To mitigate the risks associated with double-strand breaks, integrating novel CRISPR systems is crucial (Kofoed et al., 2023). BEs and PEs enable precise nucleotide changes and small insertions/deletions without inducing DSBs, dramatically reducing the risk of off-target effects and offering a superior safety profile for clinical use (Komor et al., 2016). However, PEs have higher molecular weights (∼6.3 kb) than Cas9 (∼4.2 kb), posing a significant challenge for MSC-Exos packaging capacity. This limitation may require further optimization of the ‘bait-prey’ systems or even the exploration of alternative loading strategies specifically designed for oversized cargo. The pursuit of safety must therefore be matched with innovations in delivery vehicle engineering.”

Beyond off-target effects, Cas9 immunogenicity poses a significant translational barrier, as pre-existing immunity against *Streptococcus* pyogenes Cas9 has been detected in a substantial proportion of the human population (Charlesworth et al., 2019). Chromosomal rearrangements, including large deletions and translocations, have also been documented following Cas9-mediated cleavage (Kosicki et al., 2018). Furthermore, the delivery format critically influences safety: plasmid DNA prolongs Cas9 expression and carries insertional mutagenesis risk, mRNA limits exposure duration but may still trigger innate immune responses, while ribonucleoprotein (RNP) complexes offer the shortest half-life and lowest off-target propensity (Lino et al., 2018). These considerations highlight the need to carefully optimize both the CRISPR platform and its delivery format for each therapeutic application.

The ultimate vision is an autonomous, theranostic vector that senses disease activity, delivers the appropriate genomic medicine with cellular precision, and confirms target engagement—all within a single, administrable entity. Achieving this will require interdisciplinary collaboration across vesicle biology, synthetic biology, and clinical immunology, and represents a long-term goal rather than an immediate reality. In contrast to existing reviews that primarily focus on exosome biology or genome editing technologies in isolation, this review offers an integrative, decision-oriented framework that systematically compares engineering strategies for MSC-Exos-based CRISPR delivery. By explicitly aligning technical feasibility, safety considerations, and regulatory implications, we aim to provide actionable guidance for platform selection rather than a purely descriptive synthesis.

The strategic integration of engineered MSC-Exos with the CRISPR-Cas9 system heralds a new era in the treatment of immune diseases. This review has articulated a comprehensive framework—from the detailed engineering of the vesicles for efficient cargo loading and targeted delivery, to their multifaceted mechanisms of action, and a critical assessment of the translational landscape, acknowledging both the immense potential and the significant hurdles that lie ahead. By leveraging exosomes’ innate biological properties and augmenting them with the precision of synthetic biology and gene editing, this platform holds the potential to achieve durable, targeted immune reprogramming. Realizing this potential, however, will require concerted efforts to overcome the challenges in manufacturing, safety profiling, and regulatory navigation. As the fields of vesicle biology and gene editing continue to mature, it is important to recognize that most current strategies remain at the preclinical stage, and substantial hurdles—including manufacturing standardization, long-term safety evaluation, and regulatory approval—must be overcome before clinical application becomes feasible. Nonetheless, the vision of a single-administration, curative therapy for a range of currently intractable immune diseases moves closer to reality, albeit through a path that demands rigorous scientific and clinical validation.
